# Notch3 expression in capillary pericytes predicts worse graft outcome in human renal grafts with antibody‐mediated rejection

**DOI:** 10.1111/jcmm.17325

**Published:** 2022-05-25

**Authors:** Yichun Xu‐Dubois, Panagiotis Kavvadas, Zela Keuylian, Alexandre Hertig, Eric Rondeau, Christos Chatziantoniou

**Affiliations:** ^1^ INSERM UMRS 1155 Tenon Hospital Paris France; ^2^ Public Health Assistance Publique‐Hôpitaux de Paris (AP‐HP) Tenon Hospital Paris France; ^3^ Sorbonne University Paris France; ^4^ Nephrology Department Foch Hospital Suresnes France; ^5^ Intensive Care Nephrology and Transplantation Department Tenon Hospital APHP Paris France

**Keywords:** antibody‐mediated graft rejection, kidney, Notch3, pericytes

## Abstract

Microvasculature consisting of endothelial cells and pericytes is the main site of injury during antibody‐mediated rejection (ABMR) of renal grafts. Little is known about the mechanisms of activation of pericytes in this pathology. We have found recently that activation of Notch3, a mediator of vascular smooth muscle cell proliferation and dedifferentiation, promotes renal inflammation and fibrosis and aggravates progression of renal disease. Therefore, we studied the pericyte expression of Notch3 in 49 non‐selected renal graft biopsies (32 for clinical cause, 17 for graft surveillance). We analysed its relationship with patients’ clinical and morphological data, and compared with the expression of partial endothelial mesenchymal transition (pEndMT) markers, known to reflect endothelial activation during ABMR. Notch3 was de novo expressed in pericytes of grafts with ABMR, and was significantly correlated with the microcirculation inflammation scores of peritubular capillaritis and glomerulitis and with the expression of pEndMT markers. Notch3 expression was also associated with graft dysfunction and proteinuria at the time of biopsy and in the long term. Multivariate analysis confirmed pericyte expression of Notch3 as an independent risk factor predicting graft loss. These data suggest that Notch3 is activated in the pericytes of renal grafts with ABMR and is associated with poor graft outcome.

## 1 INTRODUCTION

Antibody‐mediated rejection (ABMR) is a major cause of long‐term graft failure.^1^ Graft interstitial fibrosis, tubular atrophy and microvascular rarefaction are the common features observed in the late stage of disease. Much attention has been focused on the endothelial injury for understanding the mechanisms of antibody‐mediated graft damage and failure, because endothelial cells are the main target of donor specific antibodies (DSA), which may activate, deregulate and even cause endothelial cell death.^2^, ^3^, ^4^, ^5^, ^6^, ^7^ We have previously shown that partial endothelial to mesenchymal transition (pEndMT), evaluated by the expression of three markers fascin, vimentin and heat shock protein 47 (hsp47) in peritubular capillaries, was a highly sensitive indicator for the diagnosis of endothelial activation during ABMR, and strongly predicted poor graft outcome.^8^ Recently, we also reported that pEndMT detected in the early biopsies post‐transplant with acute tubular necrosis was associated with poor renal graft recovery.^9^ In addition, circulating anti‐Human Leukocyte Antigen (HLA) DSA were predictive of long‐term graft loss only if they associated with pEndMT in the grafts.^10^ Altogether, these results make pEndMT a mandatory intermediate step linking graft ischemia and/or harmful DSA to graft failure and suggest that expression of pEndMT markers is an interesting indicator of microvascular endothelial activation and/or injury in order to improve renal graft surveillance.

Microvasculature is constituted of endothelial cells and pericytes which crosstalk during development, maturation, stabilization and regulate together vascular tone. In pathological conditions, pericytes may amplify the response to endothelial injury and promote inflammation and fibrosis.^11^, ^12^ Activated pericytes change their phenotype and detach from endothelial cells because of an imbalance between A disintegrin and metalloproteinase with thrombospondin motifs (ADAM‐TS) and tissue inhibitor of metalloproteinase 3 (TIMP3).^13^ They will then act as myofibroblasts and produce extracellular matrix (ECM).^11^ The absence of pericytes is also detrimental because it exacerbates endothelial dysfunction and results in microvascular rarefaction and organ ischemia.^6^, ^14^ These data clearly show that pericytes play a pivotal role in maintaining the structural and functional integrity of microvasculature, whereas their activation under pathological conditions promotes inflammation and fibrosis. ABMR is a prototypical model of endothelial injury,^3^, ^15^, ^16^, ^17^ where endothelial cells could subsequently stimulate pericytes by direct contact and/or through the release of pro‐inflammatory cytokines. To date, little is known about the role of pericytes‐endothelial cell interaction in the mechanisms leading to ABMR.

The Notch receptor signalling pathway is a well‐established, cell fate‐determining system that plays a major role in the development of practically every tissue. In mammals, it consists of four members, Notch1–4, all with similar structure, consisting of a large extracellular, a small transmembrane and a transcription activating intracellular domain.^18^ Studies in experimental models of cardiac allograft transplantation suggested that Notch signalling is a major pathway promoting inflammation and that targeting the Notch pathway has therapeutic potential to prevent allograft rejection.^19^

Notch3 is generally regarded as the vascular member of the family, being expressed by vascular smooth muscle cells and regulating proper vascular development and reactivity.^20^ We have previously demonstrated that in chronic and acute kidney injury models, Notch3 is de novo expressed by suffering cells to promote renal inflammation and fibrosis and aggravate the progression of renal disease.^21^, ^22^, ^23^ Genetic manipulation inducing activation of Notch3 signalling dramatically deteriorated cell phenotype after injury, while genetic inhibition of Notch3 expression rescued renal function and structure.^23^ Thus, our previous results establish de novo activation of Notch3 as a novel pro‐inflammatory mediator which controls the phenotype of injured cell populations and promotes the progression of renal disease.

In the present study, we studied whether Notch3 expression is associated with pericyte activation during ABMR in human renal grafts and in cultured pericytes stimulated by pro‐inflammatory cytokines. Our data show that Notch3 expression is induced in pericytes during ABMR. This de novo pericyte expression of Notch3 is correlated with the expression of markers of pEndMT, as well as with graft dysfunction and proteinuria both at the time of biopsy and in the long term.

## 2 METHODS

### 2.1 Patients and samples collection

Applying a case‐control strategy, we analysed retrospectively the expression of Notch3 in 49 biopsies taken from 6 days to 6.5 years post‐transplant (median was 112 days) from 49 renal transplanted patients during 2010 to 2012, in our centre based on available tissue material. According to Banff classification,^24^, ^25^, ^26^ we included in this study 13 biopsies diagnosed with antibody mediated rejection (ABMR), and compared with 36 biopsies without ABMR including four biopsies with T‐cell‐mediated rejection (TCMR) or borderline lesions, 16 with diagnoses other than rejection (five biopsies with acute tubular necrosis, eight with graft fibrosis, three for no classified graft diseases such as minimum graft inflammation) and 16 normal biopsies taken for graft surveillance at 3 months post‐transplant. Anti‐HLA (A, B, Cw, DR, DQ and DP) DSA were detected by single antigen technology (One Lambda, Inc., Canoga Park, CA) on a Luminex platform and their strength was presented as a mean fluorescence intensity (MFI) for circulating donor specific antibodies in serum samples obtained at the time of transplantation and also that of biopsy. Routine clinical data were obtained from patient medical charts. Graft function shown by estimated Glomerular Filtration Rate (eGFR) and proteinuria were studied at the time of biopsy, plus at one and two years post‐biopsy. The demographic, clinical and morphological characteristics of patients are shown in Table 1. All patients provided informed consent to participate in the study.

**TABLE 1:** Demographic, clinical and morphological characteristics of patients

	All patients (*n* = 49)	Notch3‐ patients (*n* = 27)	Notch3+ patients (*n* = 22)	*p* (Mann–Whitney)
Recipient male sex	28/49 (57%)	19/27 (70%)	9/22 (41%)	0.038
Recipient age (years)	50.5 ± 14	50.8 ± 15	50 ± 12	0.63
Donor's age years)	52.4 ± 17	51.6 ± 16	53.4 ± 18	0.475
Deseased donor	39/49 (80%)	20/27 (74%)	19/22 (86.4%)	0.288
Donor's terminal serum creatinine (µmol/L)	82.55 ± 33.9	79.8 ± 29	86.22 ± 34	0.8154
Expanded criteria donor	16/49 (32.7%)	7/27 (26%)	9/22 (41%)	0.266
Cold ischemia time (hours)	15.3 ± 8	13.8 ± 8	17 ± 7.55	0.25
DSA at T0	19/49 (38.8%)	12/27 (44.4%)	7/22 (31.8%)	0.367
DSA at time of biopsy	26/49 (53%)	16/27 (59%)	10/22 (45.45%)	0.336
Time post Tx to biopsy (days)	370 ± 693	256 ± 463	511 ± 893	0.494
Biopsy for cause	30/49 (61.2%)	14/27 (51.85%)	16/22 (72.7%)	0.136
eGFR (MDRD) at time of biopsy (ml/min)	36.33 ± 20	43.22±19	27.9 ± 18	0.0084
eGFR (MDRD) 1 y post biopsy (ml/min)	39.1 ± 21	45.2 ± 17	31.5 ± 23	0.0358
eGFR (MDRD) 2 y post biopsy (ml/min)	35.46 ± 22	44.3 ± 17	24.5 ± 23	0.0061
ProtU at time of biopsy (g/mmol)	118 ± 202	72.3 ± 159	174.7 ± 237	0.01
ProtU 1 y post biopsy (g/mmol)	148 ± 346	87.84 ± 199	243 ± 489	0.087
ProtU 2 y post biopsy (g/mmol)	95.4 ± 224	57.8 ± 72	160 ± 359	0.93
g	0.41 ± 0.84	0.037 ± 0.19	0.86 ± 1.08	0.0004
ptc	0.55 ± 0.96	0.11 ± 0.32	1.09 ± 1.19	0.0005
i	0.3 ± 0.58	0.26 ± 0.6	0.36 ± 0.58	0.35
t	0.22 ± 0.59	0.3 ± 0.72	0.14 ± 0.35	0.58
v	0.08 ± 0.34	0	0.18 ± 0.34	0.05
ci	0.9 ± 0.96	0.8 ± 0.9	1.04 ± 1.04	0.424
ct	0.74 ± 0.9	0.63 ± 0.9	0.86 ± 1	0.456
cv	0.73 ± 0.8	0.52 ± 0.7	1 ± 0.86	0.053
c4d	0.1 ± 0.3	0.04 ± 0.2	0.17 ± 0.4	0.19

### 2.2 Immunohistochemistry of Notch3 detection and double staining with the endothelial marker CD34

Notch3 expression was detected in paraffin tissue by immunohistochemistry. Target retrieval was carried out by heating the tissue in citrate buffer. The sections were incubated overnight at 4°C with phosphate‐buffered saline containing anti‐Notch3 antibodies (gift kindly offered by Dr. Anne Joutel, INSERM UMR 1266,^27^). The immunoreactive Notch3 expression was visualized using Envision + HRP system (AEC, DAKO). For the negative technique controls, the primary antibodies were replaced by an equal concentration of mouse IgG. As sample controls, Notch3 expression was studied in 2 healthy kidneys in the vicinity of renal carcinomas. In addition, Notch3 expression was also studied in three renal grafts removed because of incurable ABMR leading to loss of graft function.

Notch3 expression in the pericytes of peritubular capillaries was semi‐quantified 3 times in a blind fashion to clinic or morphological data. Score 0: no staining; 1: <10%; 2: 10–24%; 3: 25–50%; and 4: >50% of peritubular capillaries show Notch3 pericyte staining. Grafts with a score ≥1 were considered Notch3‐positive graft. This cut‐off was defined before any statistical analysis, based on the level of Notch3 expression observed in normal kidneys. Double staining of Notch3 with endothelial marker CD34 was done according to the polink DS‐MM kit (GBI Labs).

### 2.3 Immunohistochemistry of pEndMT markers

pEndMT markers (fascin, hsp47 and vimentin expression) were detected by immunohistochemistry in paraffin tissue, assessed 3 separate times in the peritubular capillary endothelial cells blinded to the clinical and morphological data. The staining of pEndMT marker expression was semi‐quantified with the same method as described previously^8^, ^10^ for all biopsies. The highest score for any of the three markers defined the final pEndMT score. A graft with a score ≥2 (10% or more of peritubular capillary endothelial cells strongly stained) was considered pEndMT‐positive. This cut‐off was defined before any statistical analysis.

### 2.4 Notch3, pro‐inflammatory and pro‐fibrotic gene mRNA expressions in cultured human pericytes stimulated by pro‐inflammatory cytokines

The human brain vascular pericytes (HBVPs) cell line (ScienCell) was cultured in pericyte medium (ScienCell) supplied with 100 U/ml penicillin/streptomycin (Gibco) on poly‐l‐lysine coated flasks according to the manufacturer's instructions. For cytokine activation, cells were seeded on poly‐l‐lysine coated 12‐well plates. Cells were activated for 24 h with recombinant Tumour Necrosis Factor‐α (TNF‐α) (30 ng/ml), Interferon‐γ (INF‐γ) (500 U/ml) or Interleukin‐6 (IL‐6) (50 ng/ml). All reagents were purchased from Sigma‐Aldrich. Total RNA was extracted using Spin Column Total RNA Mini‐Preps Super Kit (Proteogenix). 18S rRNA was used as housekeeping gene. The primer sequences used for the different genes were as follows:

**Table:** 

InterCellular Adhesion Molecule 1 (ICAM‐1)	CCTTCCTCACCGTGTACTGG
	AGCGTAGGGTAAGGTTCTTGC
Integrin b3 (Intb3)	CAACTGGAACTTGTCAAATGAGTC
	TTAAACTGGGGTGATTCAATTTTT
Monocyte Chemoattractant Protein 1 (MCP‐1)	TTCTGTGCCTGCTGCTCAT
	GGGGCATTGATTGCATCT
Notch3	GCCAAGCGGCTAAAGGTA
	CACTGACGGCAATCCACA
Receptor for Advanced Glycation Endproducts (Rage9)	CACACTGCAGTCGGAGCTAA
	GCACAGGCTCCCAGACAC
Vascular Cell Adhesion Molecule 1 (VCAM‐1)	ACATGGAATTCGAACCCAAA
	TGTATCTCTGGGGGCAACAT

All experiments were performed in triplicate, *n* = 4 for all conditions.

### 2.5 Statistical analysis

The level of Notch3 staining in the pericytes in the patients with ABMR was compared with that in different control groups by a two‐sided T‐test and *p* was confirmed by nonparametric Mann–Whitney test. The clinical and morphological data were also compared according to the Notch3 pericyte expression status. The association of pericyte Notch3 expression, as well as that of pEndMT marker expression in the microvasculature endothelial cells with clinical data, MFI of anti‐HLA DSA, Banff histological scores, graft function and proteinuria at different time points were evaluated by Spearman's rank‐order correlation analysis (Rho coefficients are shown). Kaplan–Meier curves were drawn according to Notch3, or to pEndMT status to study the influences of endothelial or pericyte injury/activation on graft survival. Graft survival time was calculated from the date of biopsy to the date of graft loss (in patients for whom multiple biopsies were performed, only the latest biopsy was taken into account for this analysis). To further confirm the independent association of Notch3 or pEndMT marker expression with graft survival, Cox regression analysis was used to adjust with the most important risk factors for graft loss. Here, the presence of anti‐HLA DSA was subgrouped as follows: MFI < 500, 500–999, 1000–2999, 3000–4999, 5000–9999 and ≥10,000. The level of significance was set up at *p* < 0.05. Data analysis was performed using the Stata version 8.

## 3 RESULTS

### 3.1 Notch3 is de novo expressed in the pericytes of peritubular capillaries in grafts with ABMR

**FIGURE 1:**
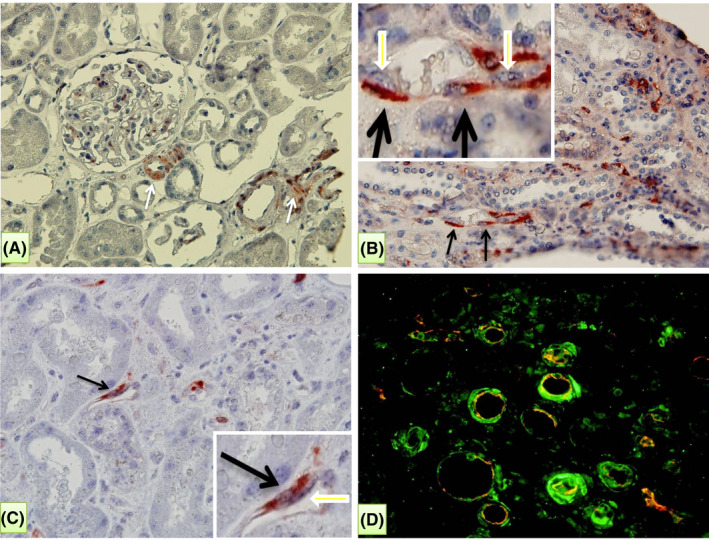
Expression of Notch3 in normal grafts (A), and in grafts with antibody‐mediated rejection (ABMR) (B–D). (A) In normal kidneys, expression of Notch3 was mainly found to vascular smooth muscle cells (white arrows). No staining was found in peritubular capillaries. (B, C) During ABMR, a strong expression of Notch3 was observed in pericytes of peritubular capillaries (black arrows) closely attached to the endothelial cells (see inserts in the upper left and lower right corners for B and C, respectively). (D) Double staining with the vascular endothelial marker CD34 (red) and Notch3 (green) indicated that Notch3 was closely attached to and around the capillary endothelial cells in the biopsies with ABMR

**FIGURE 2:**
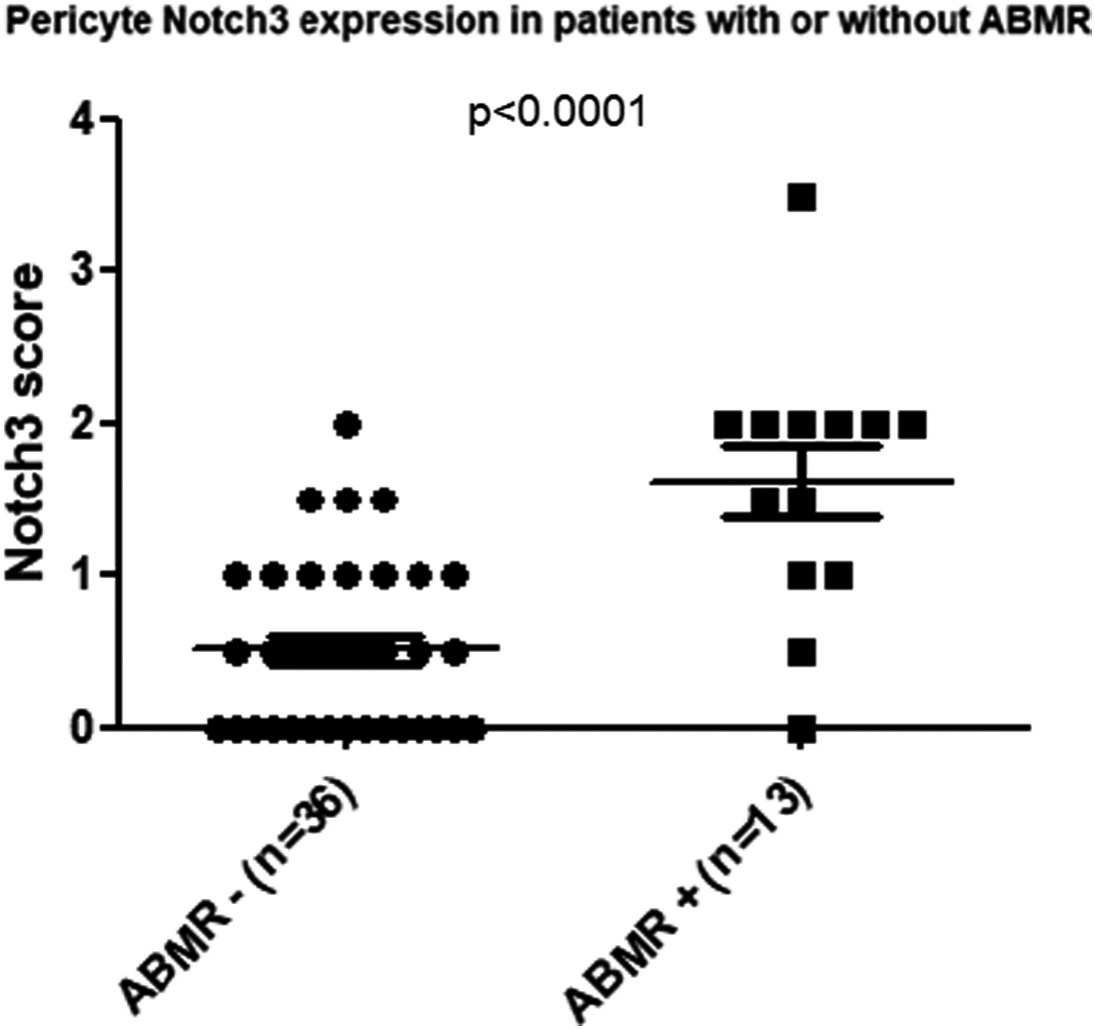
Notch3 expression evaluated by immnunohistochemistry in biopsies from patients with or without ABMR. To note that significantly higher levels of Notch3 pericyte expression was found in renal grafts with antibody‐mediated rejection (ABMR) compared to those without or in grafts with either no lesion or lesions due to other causes (*n* = 13 for ABMR, *n* = 36 for other, *p* < 0.0001)

In normal kidneys, Notch3 expression was observed mainly in vascular smooth muscle cells and in some glomerular mesangial cells (white arrows, Figure 1A). No staining could be found in the pericytes of peritubular capillaries. In the grafts with ABMR, a strong Notch3 expression was detected in numerous pericytes closely attached to the endothelial cells of peritubular capillaries (black arrows, Figure 1B,C). Double staining with the vascular endothelial marker CD34 (in red), confirmed that the de novo expression of Notch3 (in green) is mainly in pericytes of the peritubular capillaries, because it is found in cells adjacent to CD34 expressing cells (Figure 1D). This de novo expression of Notch3 was not observed in pericytes of protocol biopsies for the graft surveillance with normal graft function and without morphological anomalies, neither in the grafts with the pathologies other than ABMR, such as TCMR. Semi‐quantification showed that the level of Notch3 expression in pericytes was significantly higher in the grafts with ABMR compared with those without (*p* < 0.0001, *n* = 13 and 36 biopsies, for AMBR vs other, respectively, Figure 2).

### 3.2 Association analysis of Notch3 expression in pericytes with clinical data, graft lesions (Banff scores) and expression of markers of pEndMT

Table 2 shows a significantly higher level of inflammation in graft microcirculation in Notch3‐positive grafts group compared with those in Notch3 negative grafts group (1.09 ± 1.19 vs 0.11 ± 0.32, *p* = 0.0005 for peritubular capillaritis (ptc) score; 0.86 ± 1.08 vs 0.037 ± 0.19, *p* = 0.0004 for glomerulitis (g) score). Using Spearman's rank‐order correlation analysis, the score of pericyte Notch3 expression was significantly correlated with ABMR‐related lesions (rho = 0.57, *p* < 0.0001 for the correlation with microcirculation inflammatory ptc and g scores), but not with TCMR associated lesions such as i or t scores (Table 2).

**TABLE 2:** Correlations of Notch3 expression in pericytes and of pEndMT markers in endothelial cells of peritubular capillaries with the clinic and morphological lesions

	With Notch3		With pEndMT	
Correlation of	Rho	*p*	Rho	*p*
g	0.57	<0.0001	0.664	<0.0001
ptc	0.57	<0.0001	0.73	<0.0001
i	0.21	0.13	0.48	0.0004
t	0.024	0.87	0.32	0.02
v	0.31	0.02	0.34	0.016
cg	0.31	0.028	0.465	0.0008
ci	0.18	0.2	0.454	0.0011
ct	0.13	0.35	0.5	0.0002
cv	0.3	0.035	−0.0008	0.996
c4d	0.22	0.16	0.32	0.045
pEndMT	0.35	0.012		
Cold ischemia time	0.15	0.3	0.38	0.0061
eGFR at time of biopsy	−0.45	0.0007	−0.39	0.0051
eGFR 1 year after biopsy	−0.346	0.0128	−0.6	<0.0001
eGFR 2 years after biopsy	−0.428	0.0017	−0.49	0.0004
Proteinuria at time of biopsy	0.49	0.0003	0.468	0.0008
Proteinuria 1 year after biopsy	0.316	0.0368	0.1214	0.4437
MFI highest to DSA HLA i	−0.184	0.19	0.2	0.166
MFI highest to DSA HLA ii	0.06	0.65	0.46	0.0007
MFI highest to DSA HLA i or ii	0.01	0.94	0.456	0.0009

As expected, the microcirculation inflammatory score was associated with graft dysfunction at the time of biopsy (rho = −0.38, *p* = 0.007 for the Spearman's rank‐order correlation with g score; rho = −0.443, *p* = 0.0014 with ptc score) and at 2 years post‐biopsy (rho = −0.413, *p* = 0.0039 with g score; rho = −0.438, *p* = 0.0021 with ptc score). However, graft dysfunction was not associated with TCMR‐related lesions such as i and t score neither for the eGFR at the time of biopsy (rho = −0.14, *p* = 0.33, for i score; and rho = −0.06, *p* = 0.68 for t score); nor for at 2 years after biopsy (rho = −0.045, *p* = 0.76 for i score; rho = 0.1, *p* = 0.5 for t score).

We found that the patients with grafts positive to Notch3 in pericytes had a significantly lower graft function at time of biopsy (eGFR:27.9 ± 18 vs 43.22 ± 19 ml/min, *p* = 0.0084) as well as at 2 years after biopsy (24.5±23 vs 44.3±17, *p* = 0.0061) compared with those without (Table 2). The level of Notch3 expression in pericytes was significantly correlated with graft dysfunction at time of biopsy (rho = −0.45, *p* = 0.0007), as well as at 2 years after biopsy (rho = −0.428, *p* = 0.0017, Table 2). Notch3 score was also significantly correlated with the level of proteinuria at time of biopsy and 1 year after (rho = 0.49, *p* = 0.0003 and rho = 0.316, *p* = 0.0368, respectively, Table 2).

Next, we evaluated the expression of pEndMT markers in the grafts of these patients (Suppl Figure 1). As expected, we found that the pEndMT was strongly correlated with ABMR‐related lesions such as g (rho = 0.664, *p* < 0.0001), ptc scores (rho = 0.73, *p* < 0.0001) and was associated with graft dysfunction at time of biopsy and up to 2 years after biopsy (Table 2). Interestingly, we found a significant and positive correlation of the score of Notch3 expression in pericytes with the pEndMT score: rho = 0.35, *p* = 0.012 (Table 2). This correlation suggests a concomitant activation of these two types of capillary structural cells in renal grafts during ABMR.

We confirmed also our previous findings^8^, ^9^, ^10^ that the pEndMT score was associated with the cold ischemia time (rho = 0.38, *p* = 0.0061), and with the strength of immune‐dominant anti‐HLA class II DSA (measured by MFI) at the time of biopsy (rho = 0.46, *p* = 0.0007). However, correlations of Notch3 expression in pericytes with the cold ischemia time or with MFI for anti‐HLA class I or II were not observed (Table 2).

### 3.3 Notch3 expression in pericytes and expression of pEndMT markers in endothelial cells are two independent risk factors predicting renal graft loss

**FIGURE 3:**
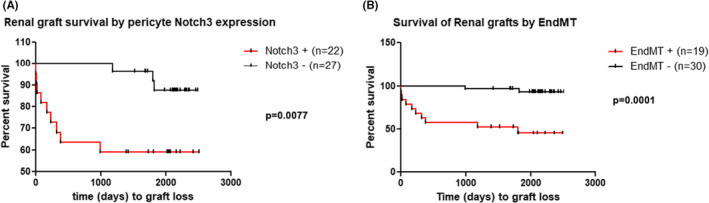
Kaplan–Meier curves for allograft survival rate analysis according to pEndMT markers expression in endothelial cells (B). Wilcoxon (Breslow) test for equality of survivor functions: Chi2(1) = 7.09, *p* = 0.0077 and Chi2(1) = 7.09, *p* = 0.0077, for (A) and (B), respectively

During more than 5 years follow‐up (Median follow‐up was 5.9 years), 11 among 49 studied patients (22.45%) lost their grafts. When Kaplan–Meier graft survival curves were plotted according to Notch3 expression in pericytes of peritubular capillaries, the group of patients with Notch3 expression lost their grafts earlier (mean time of 0.7 vs 4.4 years) and more frequently than those without (*p* = 0.0077) (Figure 3A). When Kaplan–Meier graft survival curves were plotted according to expression of pEndMT markers in endothelial cells of peritubular capillaries, we observed a similar result: pEndMT‐positive grafts were lost earlier (mean time of 1.3 vs 3.8 years) and had a significantly lower graft survival rate (*p* = 0.0001) (Figure 3B).

The independent predictive value of Notch3 expression in the pericytes for graft loss was confirmed by the COX regression model with a HR of 6, *p* = 0.018 (95% IC: 1.35–27) after adjustment with the most important risk factors for graft loss such as the presence of DSA against HLA class I or II at time of biopsy, time since transplantation and the presence of interstitial fibrosis (ci score) in the graft at time of biopsy (Table 3, model 1). Similarly, expression of pEndMT was also an independent predictive marker for graft loss through the COX regression model with a HR of 13.5, *p* = 0.007 (95% IC: 2‐89, Table 3, model 2).

**TABLE 3:** Independent risk factor analysis of graft loss by COX model (11 graft loss)

Risk factor for graft loss	HR	*p*	IC
Model 1			
Notch3‐positive	6.03	0.018	1.35–27
DSA anti‐HLA i or ii	1.27	0.34	0.78–2.06
Biopsy time since Transplant	1.65	0.003	1.18–2.3
Graft fibrosis (ci score)	2.5	0.012	1.22–5.09
Model 2			
pEndMT‐positive	13.5	0.007	2.07–89
DSA anti‐HLA i or ii	0.9	0.68	0.52–1.5
Biopsy time since Transplant	1.33	0.095	0.95–1.9
Graft fibrosis (ci score)	2.4	0.012	1.2–4.9

### 3.4 Notch3 expression is induced in cultured human pericytes following stimulation by pro‐inflammatory cytokines IL‐6 and INF‐γ

**FIGURE 4:**
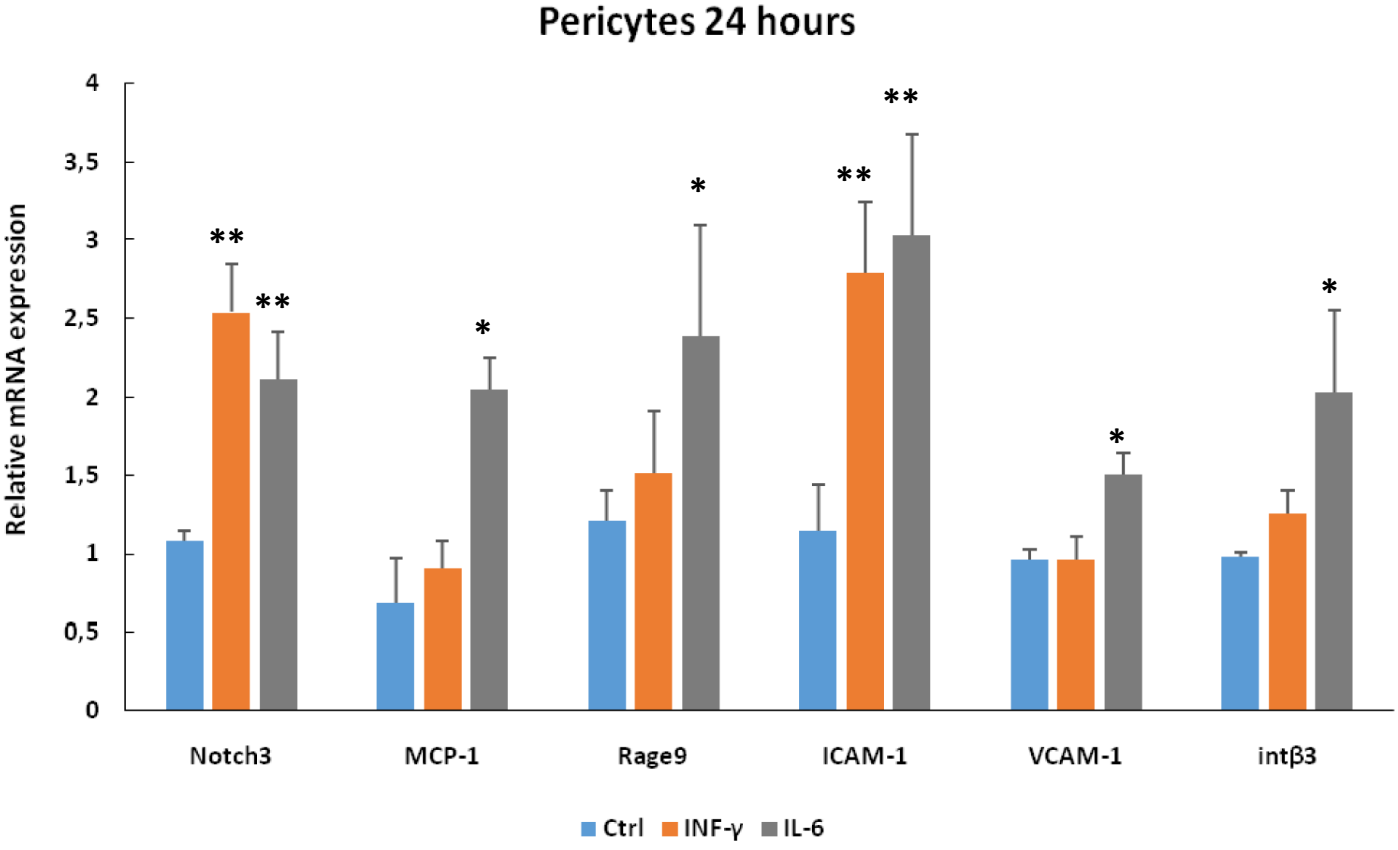
Human pericytes cultured under control conditions or after administration of IL‐6 or INFγ. To note that both cytokines strongly induced Notch3 expression. This increase was accompanied by concomitant increase of several inflammatory and fibrotic markers, including MCP‐1, Rage9, ICAM‐1 and VCAM‐1 and the pro‐migratory integrin β3. (*p < 0.05; **p < 0.01, *n* = 4)

To examine whether Notch3 is upregulated in pericytes in conditions that mimic the pro‐inflammatory environment in rejected grafts, we incubated human pericytes with INF‐γ or IL‐6, two major pro‐inflammatory cytokines, known to activate pericytes towards to a fibroblast phenotype. Addition of IL‐6 treatments increased Notch3 mRNA expression along with several ‘classical’ pro‐inflammatory and pro‐fibrotic genes such as MCP‐1, Rage9, ICAM‐1, VCAM‐1 and integrin β3 (Figure 4), while INF‐γ increased mainly Notch3 and ICAM‐1 expressions. These in vitro results indicate that percicytes, when activated under the effect of pro‐inflammatory cytokines and start to change their phenotype, strongly express Notch3.

## 4 DISCUSSION

In the present study, we showed a de novo expression of Notch3 in pericytes during ABMR. Notch3 expression was associated with ABMR‐related histological lesions (g and ptc scores) and with the expression of endothelial activation markers pEndMT, but not with TCMR‐related lesions (such as i and t scores). This probably reflects a biological response of pericytes in the environment of an immune‐mediated endothelial injury within capillaries. The significant correlation of Notch3 expression in pericytes and of pEndMT markers in endothelial cells with proteinuria and renal graft dysfunction at the time of biopsy, as well as in the long term, strengthens the idea that microvasculature injury largely contributes to graft destruction and function loss.

The expression of Notch3 in activated pericytes during ABMR is a novel and interesting finding. Notch3 is a mammalian heterodimeric transmembrane receptor which under normal conditions is almost exclusively expressed by vascular smooth muscle cells and plays a major role in the formation of the structure of small resistance vessels during development. Notch3 mutations cause CADASIL (Cerebral Autosomal Dominant Arteriopathy with Subcortical Infarcts and Leukoencephalopathy), a hereditary autosomal dominant disease, characterized by progressive degeneration of smooth muscle cells of small brain vessels, leading to dementia and death.^27^, ^28^, ^29^ Using mice lacking expression of Notch3, we demonstrated that Notch3 plays an important role in the maintenance of normal tone of the renal resistance vessels.^20^ The renal vasculature of these animals did not contract properly to acute administration of vasoactive agents such as angiotensin II or norepinephrine because of a deficient myogenic response. The lack of a proper myogenic response was attributed to an immature formation of the vascular wall structure. This deficiency has important long‐term implications because mice lacking Notch3 display altered blood pressure increase and develop a severe cardiac and renal phenotype when hypertension is induced chronically.^20^, ^30^

In adulthood, Notch3 is still expressed in renal resistance vessels, but it is rather ‘silent’ under normal conditions. In contrast, under pathological conditions Notch3 can be de novo expressed by the suffering cells to promote disease. Thus, hypoxia‐induced proliferation of vascular smooth muscle cells, known to be of critical importance in the progression of pulmonary arterial hypertension, was favoured in response to Notch3 signalling.^31^ Similarly, in several models of renal disease such as glomerulonephritis, unilateral ureteral obstruction or ischemia/reperfusion injury we found that Notch3 was expressed in the suffering cells (podocytes, tubular interstitial or proximal tubular, respectively) and induced renal inflammation, proliferation and fibrosis. Mice lacking Notch3 were significantly protected against the progression of renal disease in these models, whereas mice genetically modified to express a continuously activated Notch signalling were progressing faster towards loss of renal function and structure.^21^, ^22^, ^23^

Several studies indicate a major interaction between Notch3 and inflammation. Notch3 and NF‐κB pathway crosstalk has been extensively studied in T‐cells and smooth muscle cells. Overexpression of N3ICD (the intracellular domain of Notch3 and effector of Notch signalling) in T‐cells activates NF‐κB signalling^32^ and the mechanism driving this activation is related with degradation of the inhibitory IkBα and nuclear translocation of NF‐κB subunits.^33^ We have shown that when Notch3 is transfected in renal tubular cells induces the expression of MCP‐1, RANTES and cell proliferation,^21^ and that infection of podocytes with Notch3‐carrying viruses induces NF‐κB expression.^22^ In contrast, mice lacking Notch3 gene expression failed to activate NF‐κB after renal ischemia/reperfusion injury.^23^ Furthermore, Notch3 and NF‐κB can form a positive feedback loop where Notch3 stabilizes NF‐κB subunits which in turn bind to the Notch3 promoter, activating its transcription and sustaining a pro‐inflammatory environment.^23^ Our present results shown in Figure 4 clearly indicate that pericytes in an inflammatory environment change phenotype and express Notch3 along with several markers of cellular stress.

Alternatively, Notch3 activation can be also associated with cell structural changes towards a myofibroblast phenotype. Myofibroblast origin in the renal interstitium is a topic of debate for many years. Several cell populations, among them pericytes, have been proposed to transdifferentiate into myofibroblasts during kidney disease.^11^ Whether this transdifferentiation leads to fully functional myofibroblasts and the actual extent of their contribution to renal fibrosis is still under debate. However, it appears that Notch3 affects the phenotype of cell populations possibly *via* reactivation of developmental mechanisms. Thus, Notch3 was used as a marker of endothelial to mesenchymal transition during atherosclerosis,^34^ and in hepatic stellate cell activation and transdifferentiation to myofibroblasts.^35^ In agreement with this notion, previous studies in our lab showed that transfection of podocytes with Notch3 altered phenotype and induced cell proliferation contributing and accelerating the progression of glomerular disease.^22^ Overall, although a direct link between myofibroblast activation and Notch3 signalling has yet to be established in vivo, Notch3 appears to affect multiple cell populations involved in renal disease, inducing phenotypic alterations that can compromise normal parenchyma and, in long term, renal function.

In the context of ABMR, a de novo expression of Notch3 in pericytes reflects Notch3 signalling activation in these cells. It is possible that this activation can improve pericyte survival after injury. However, pericyte activation can also exacerbate renal graft microvasculature injury and alter local oxygen diffusion through capillary remodelling with cell proliferation and hypertrophy. Active pericytes can detach from endothelial cells which creates instability of capillaries and ultimately results in capillary rarefaction, graft chronic ischemia and failure. Detached pericytes can also migrate into the interstitium and become myofibroblasts to produce extracellular matrix promoting thus graft fibrosis.

In studies like the present, which are based on patients’ functional data and biopsies, it is difficult to define the exact cellular and molecular mechanisms of Notch3 signalling activation in grafts during ABMR. The lack of a statistically significant association between Notch3 expression in pericytes with DSA at time of biopsy or with cold ischemia time during kidney transplantation, suggests an indirect effect of these two potential triggers for Notch3 pericyte expression during ABMR. Nevertheless, we cannot exclude the local ischemia at time of biopsy because of ABMR as a potential cause for pericyte activation and Notch3 expression. The observation that DSA and graft cold ischemia time were significantly correlated with the level of pEndMT (also reported previously^8^, ^9^, ^10^), suggests a more direct effect of these two factors for endothelial activation. Interestingly, in the present study, we found a significant association of the expression of Notch3 in pericytes with the markers of endothelial activation. In addition, the fact that Notch3 expression in pericytes was also significantly associated with microvascular inflammatory scores g and ptc, supports a possible paracrine role of cytokines to induce Notch3. In agreement with this hypothesis, exposure of cultured human pericytes to cytokines such as IL‐6 induced expression of Notch3 concomitant to several genes promoting inflammation, fibrosis and cell phenotype alterations (Figure 4). Taking together, these results suggest rather an effect through an inflammatory cytokines release from active endothelial and/or microvascular infiltrating cells during ABMR to promote Notch3 expression and pericyte activation and/or phenotype change. Since biopsies are ‘pictures’ of a given moment, it is difficult to know what event occurs first. It is possible that local pro‐inflammatory signals affect in parallel both endothelial cells and pericytes altering their normal, physiological phenotype towards an activated one. Thus, in the case of endothelial cells, this inflammatory activation leads to the expression of pEndMT markers, whereas in the case of pericytes to the expression of Notch3. Since there is a cell–cell communication, it is probable that these events are interact and create a loop in which activated endothelial cells promote a fibroblast‐like phenotype of pericytes and vice versa activated pericutes favour pEndMT, leading with the time to renal dysfunction and graft rejection. In agreement to this hypothesis, a positive correlation of the Notch3 expression with the pEndMT markers was observed (Table 2). This correlation suggests a concomitant activation of these two types of capillary structural cells in renal grafts during ABMR.

In conclusion, we report for the first time that Notch3 is abnormally expressed in pericytes of renal grafts with antibody‐mediated rejection. This expression is significantly correlated with the microcirculation inflammation and with the expression of markers of endothelial injury. In addition, Notch3 expression is significantly associated with graft dysfunction and proteinuria at the time of biopsy and in the long term.

## CONFLICT OF INTEREST

The authors confirm that there are no conflicts of interest.

## AUTHOR CONTRIBUTIONS

**Yi‐Chun Dubois‐Xu:** Conceptualization (equal); Formal analysis (lead); Investigation (lead); Methodology (lead); Validation (lead); Writing – original draft (equal). **Panagiotis Kavvadas:** Conceptualization (supporting); Formal analysis (supporting); Investigation (supporting); Methodology (supporting); Validation (supporting); Writing – original draft (supporting). **Zela Keuylian:** Investigation (supporting); Methodology (supporting); Validation (supporting). **Alexandre Hertig:** Conceptualization (supporting); Formal analysis (supporting); Methodology (supporting); Validation (supporting); Writing – original draft (supporting). **Eric Rondeau:** Conceptualization (supporting); Formal analysis (supporting); Methodology (supporting); Resources (supporting); Supervision (supporting); Validation (supporting); Writing – original draft (supporting). **Christos Chatziantoniou:** Conceptualization (equal); Funding acquisition (lead); Project administration (lead); Resources (lead); Supervision (supporting); Validation (supporting); Writing – original draft (equal).

## Supporting information

Figure S1
