# Supplementary figures and images for: Notch3 expression in capillary pericytes predicts worse graft outcome in human renal grafts with antibody‐mediated rejection
Source: J Cell Mol Med. 2022 May 25;26(11):3203–12. doi: 10.1111/jcmm.17325 (PMC9170800; doi:10.1111/jcmm.17325)

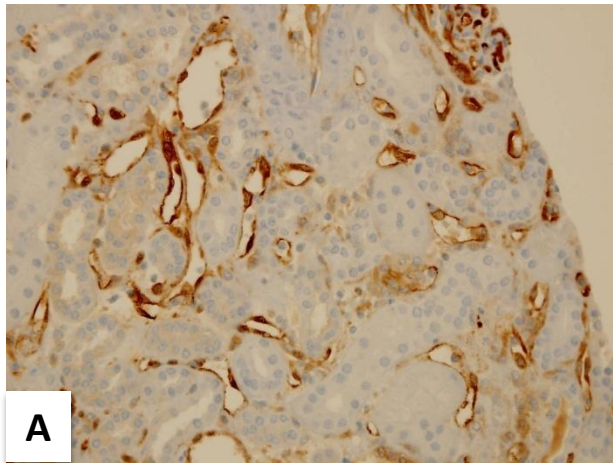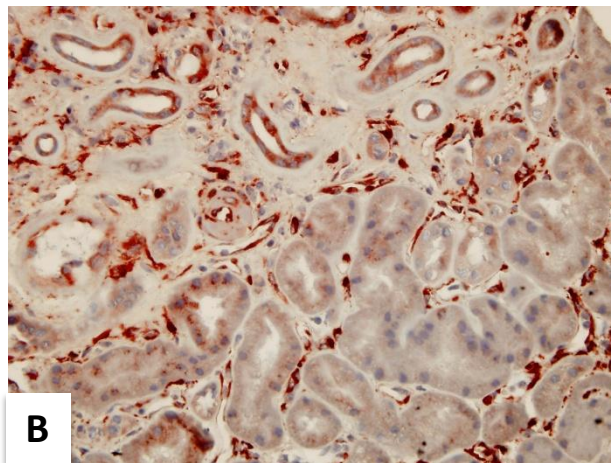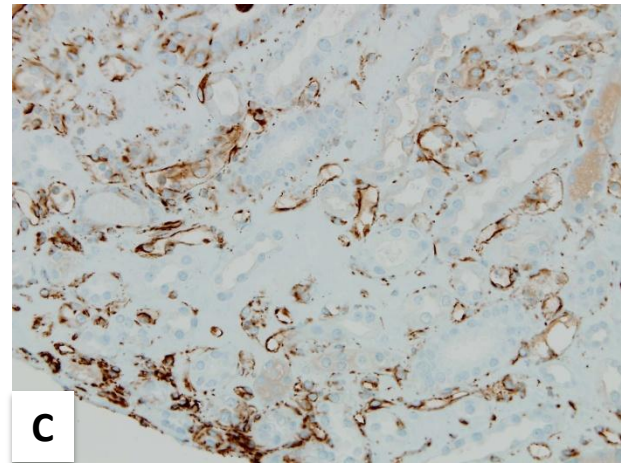

**Supplementary Figure 1**

Supplement: Supplementary file 1 — Figure S1 [file JCMM-26-3203-s001.pdf]
